# Assessing the Use of Wearable Mobile-Monitoring Devices Among Individuals With Serious Mental Illness: Qualitative Acceptability and Feasibility Study

**DOI:** 10.2196/85087

**Published:** 2026-05-15

**Authors:** Aubrey M Freitas, Jesus G Chavez, Melissa Chinchilla, Ronald Calderon, Stephanie Chassman, Lauren Hoffmann, Alexander S Young

**Affiliations:** 1 Mental Illness Research, Education, and Clinical Center, VA Greater Los Angeles Healthcare System, U.S. Department of Veteran Affairs, 11301 Wilshire Blvd, Los Angeles, CA, 90073, United States, 1 (310) 478-3711; 2 Department of Psychiatry and Biobehavioral Sciences, Semel Institute for Neuroscience and Human Behavior, University of California Los Angeles Geffen School of Medicine, Los Angeles, CA, United States

**Keywords:** serious mental illness, wearable devices, mobile sensing, wearables, patient-generated health data

## Abstract

**Background:**

Serious mental illness (SMI) is difficult to treat for various reasons, such as rapid changes in symptoms, comorbid health conditions, long gaps between provider visits, and additional societal barriers experienced by this population. Wearable mobile-sensing devices can be used to passively collect valuable patient-generated health data, such as daily step count, heart rate variability, sleep information, and other health-related behaviors, which could inform and improve treatment for individuals with SMI. Wearable health devices have become more economically accessible, providing promise for the possibility of their implementation in health care. However, more information regarding how individuals with SMI perceive and interact with these devices is needed.

**Objective:**

This study aimed to assess the acceptability and feasibility of using wearable mobile-sensing devices to improve treatment outcomes for Veterans with SMI. In addition, we were also interested in learning if privacy concerns would influence acceptability of devices, specifically surrounding location tracking and health information sharing, as well as assessing other barriers to device use.

**Methods:**

Qualitative interviews were conducted with participants who had been using a wearable health and fitness tracker for at least 2 weeks to explore their thoughts and perceptions of these devices. A total of 15 Veterans diagnosed with a SMI participated in interviews. Both thematic analysis and rapid qualitative analysis approaches were used to uncover findings in key domains and emergent themes.

**Results:**

Wearable fitness trackers allowed participants to conveniently monitor various aspects of their physical and mental health, provided a greater understanding of their overall well-being, and motivated them to reach personal health goals. Individuals were open to sharing their personal health information collected from the devices with providers to improve their health care treatment and expressed no privacy concerns surrounding data tracking or the device’s global positioning system that monitors physical location. Participants experienced some technological challenges with using the fitness trackers, as well as the device’s accompanying cell phone app. Furthermore, participants expressed difficulties in understanding and interpreting the health data that was collected from the health and fitness trackers. Greater ongoing technological support, in addition to physical device adjustments to enhance comfort and usability, were suggested ways of improving overall user experience.

**Conclusions:**

Participants with SMI in this sample were accepting of wearable mobile-monitoring devices and believe it is feasible to incorporate these fitness trackers into their daily lives. Furthermore, participants in this sample expressed no privacy concerns regarding location tracking or the sharing of health information collected from these devices with providers. Patient-generated health data collected from these devices may offer valuable information that could be used to inform health care treatment for this population.

## Introduction

### Overview

Serious mental illnesses (SMIs) impact nearly 15.4 million adults [1], are challenging to treat, and require years of monitoring and adjustments in treatment. Diagnoses of SMI have been linked to additional life challenges, including job loss [2], homelessness [3], incarceration [4], hospitalization [5,6], or suicide [7]. Clinician visits are infrequent, and illness exacerbations often occur with no clinician awareness in real time, leaving no opportunity to adjust treatment. Methods are needed for monitoring mental health and behavioral status of individuals with SMI to support outreach, care coordination, and treatment. Behavioral phenotypes have been developed from mobile data, and wearable devices, which are now common in the population and are well-suited to produce these data and support clinical services. However, it is not yet clear whether people with SMI will accept and are interested in engaging with wearable devices and mobile monitoring to support care delivery.

Individuals with SMI experience several health comorbidities [8], like cardiovascular disease [9], and have higher mortality rates than the general population [10], with some estimating about 2 to 3 times higher [11]. In addition, SMI has been associated with high financial burdens on society through increased emergency service use [12] and has been linked to high rates of disability [13]. Medication is a common treatment for SMI symptoms; however, complex medication regimens present challenges to adherence for patients, and many antipsychotic medications are associated with adverse side effects that may exacerbate or create additional health challenges [14]. Furthermore, this population also experiences additional and systemic barriers, such as stigma, poor coordination of treatment, and lack of interdisciplinary care for physical and mental health symptoms, which can contribute to mistrust in health care systems [15]. Exploring the ways mobile-sensing devices can be used to benefit treatment outcomes may address multiple overlapping barriers, risks, and rapid, unpredictable changes in symptoms.

### Prior Work

Past research has shown both increased use of and favorable attitudes toward mobile devices, particularly cell phones, among individuals with SMI [16,17], including Veterans with SMI [18], making the possibility of integrating mobile-monitoring devices and their partner cell phone apps into current health care treatments feasible for this population. Incorporating mobile health interventions via smartphones can improve treatment outcomes [19] and intervention implementation through accessible web-based therapies, leading to improvements in SMI symptoms, reductions in hospitalizations, and increases in social connectedness [20]. In addition, the use of cell phone apps has been found to increase patient knowledge of their health conditions [21], and this additional information may be particularly helpful in allowing individuals with SMI to advocate for their needs and monitor symptoms, as well as give providers more insight into their overall health, which could improve treatment planning and care coordination.

Research conducted among Veterans with SMI shows that remote sensing devices can be used to effectively monitor sleep and physical activity data [22]; however, while this research supports the efficacy of using these devices partnered with intensive support, further study is needed to examine their effectiveness when incorporated into usual care. Changes in health behaviors have been found to be correlated with the onset of psychiatric symptoms, such as abnormal sleep patterns [23] and changes in physical activity levels [24] before experiencing a relapse or a re-emergence of positive symptoms. Therefore, by monitoring these and similar health behaviors through wearable mobile-monitoring devices, patient-generated health data (PGHD) could potentially be used to predict relapses in individuals with SMI. Furthermore, if patients were able to self-monitor their health behaviors with wearable devices, they may be able to seek assistance before a relapse occurs. Additionally, if the health information from wearable devices is shared with patients’ providers, providers can track behavior changes between medical visits, and proactively reach out to patients, and, if needed, adjust treatments to prevent a lapse in symptoms. Research in this area found that clinicians are open to implementing the use of mobile-sensing technology into health care treatment [25,26]. Similarly, individuals with SMI are open to the idea of self-monitoring technology [27]. Further study is needed to better assess potential concerns of mobile-monitoring devices among this population surrounding privacy, location tracking, sharing PGHD with clinicians, as well as discovering ways to improve the devices for individuals with SMI.

### Objective

In this study, we used qualitative methods to examine the acceptability and feasibility of using mobile-sensing technology to inform and improve health care outcomes for individuals with SMI. We were interested in understanding whether participants found PGHD useful and potential barriers to data use and integration in their health care treatment. This study expands our knowledge of the thoughts and perceptions of individuals with SMI toward mobile-sensing technology and incorporating wearables into their daily lives, which can inform future research exploring the integration of PGHD collected from these devices in a clinical setting to improve treatment outcomes.

## Methods

### Recruitment

This study served as a qualitative arm of a larger pilot study [28] exploring how passive monitoring through a mobile-sensing cell phone app can be used to improve health care treatment and outcomes for individuals with SMI. To be eligible to participate, Veterans needed to receive care at the Greater Los Angeles Department of Veterans Affairs (GLA VA) and have received a SMI diagnosis of either schizophrenia, schizoaffective disorder, or bipolar disorder, or have received a diagnosis of posttraumatic stress disorder (PTSD) and be on a qualifying antipsychotic medication. Research staff screened patients’ records using the Department of Veterans Affairs (VA’s) electronic health record system, and eligible participants were recruited via letter and follow-up phone calls. For the larger study, a total of 96 participants (n=96) were enrolled (535 Veterans were approached, 197 were not interested, 229 were unable to contact, 13 were not eligible due to an early version of the project’s app being incompatible with Android smartphones).

### Study Design

Individuals who participated in qualitative interviews were randomly selected out of the total 96 Veterans in the larger arm of the project to participate in the qualitative portion of the study using wearable mobile-monitoring devices. A total of 15 Veterans (n=15) were approached, and all consented to be interviewed. These participants were provided a wearable health and fitness tracking device (a Fitbit watch, either an Inspire 2 or Inspire 3 version). Fitbit devices were deemed appropriate for this project as they assess the study’s domains of interest (eg, stress, sleep, and physical activity), are compatible with both Android and iPhone cellular devices, are relatively affordable, and are market-leaders for mobile sensing devices. At the time of the interviews, all participants had been using their wearable device for a period ranging from 2 weeks to 1 month.

All Fitbit devices come with an accompanying app that displays the user’s longitudinal health data collected from the wearable device. The app also provides access to mindfulness exercises, physical fitness routines, and allows users to input additional health information, such as food and water intake. Upon providing individuals with their watch, research staff downloaded the Fitbit app to participants’ cell phones and briefly demonstrated how the app can be used along with the wearable device itself.

Semistructured interviews were conducted by research staff. Three interviewers (AF, LH, and RC) from the study team interviewed participants individually using a semistructured interview guide. Two interviewers (AF and LH) self-identified as female and one as male (RC). Two interviewers (LH and RC) were employed at the GLA VA as qualitative analysts and held master’s-level degrees in experimental psychology and social work, and one (AF) was employed as a research coordinator and held a bachelor’s degree in psychology. All interviewers had training in qualitative methods, previous experience conducting interviews, and prior involvement working with Veterans with SMI at GLA VA.

The development of the interview guide was informed by the Technology Acceptance Model [29] and focused on perceptions of individuals with SMI toward wearable mobile-sensing devices. Interviews focused on the acceptability, feasibility, perceived usefulness, and ease of use of wearable mobile-monitoring devices for this population. In addition, interviews probed how these devices and accompanying phone app could be improved to enhance usability and overall experiences for participants. The interview guide was pilot tested by research staff before data collection and then adjusted to fit the desired length of the interviews.

Interviews lasted between 15 and 30 minutes and were conducted either in person or over the phone in private office spaces with only the interviewer and participant present. All interviews were audio-recorded and then professionally transcribed. Interviews were conducted until saturation was achieved. Saturation was defined as the point at which no new themes emerged from 3 consecutive interviews. To assess saturation, transcripts were reviewed iteratively by the research team to track the emergence of new themes and codes. After three successive interviews produced no additional themes, data collection was discontinued.

### Analytic Approach

Rapid qualitative analysis [30] was used to identify prominent findings established to be informative of the perceptions of individuals with SMI toward wearable mobile-sensing devices. A rapid qualitative analysis approach to reviewing interview transcripts was deemed appropriate as the primary goal was to gain overall feedback from Veterans, as well as elicit insights to targeted questions designed to broaden our understanding of the experiences of participants with SMI when using these devices. Four members of the research team (AF, JC, LH, and RC) developed a draft codebook based on thematic analysis, including a priori codes derived from the qualitative interview guide. Through iterative review of transcripts, the research team identified and incorporated emergent themes, refining the codebook to reflect both predefined and data-driven themes [31]. Two team members (AF and JC) then independently coded the same 2 transcripts using Atlas.ti 24 software (Lumivero) and met afterward to compare and discuss the results and modify the codebook based on their findings. Any coding discrepancies were reconciled by consensus. These two authors (AF and JC) then separately coded identical transcripts once more using the refined codebook and met again to compare coding and establish strong intercoder reliability. No discrepancies in coding were found. All remaining interviews were then coded by AF and JC. Findings were organized using emergent themes from interviews.

### Ethical Considerations

This research was approved by the Veterans Health Administration’s Institutional Review Board (2018‐020118). Written informed consent was obtained from participants upon enrollment into the parent study, and verbal consent was also obtained from participants before the start of each qualitative interview. This study used deidentified data to safeguard participant information. Participants received US $10 in compensation for completing qualitative interviews. Reporting of qualitative research in this manuscript is aligned with guidelines for qualitative research in informatics [32].

## Results

### Sample

Ages of participants ranged from 34 to 69 years with a mean age of 51.7 (SD 11.9) years. Four of the participants self-identified as women and 11 participants self-identified as men. Five participants were diagnosed with a schizophrenia spectrum disorder, 6 were diagnosed with bipolar disorder, and 4 were diagnosed with PTSD. In addition, 8 participants held diagnoses for 2 qualifying SMIs, 6 with a combination of bipolar disorder and PTSD, and 2 with a combination of a schizophrenia spectrum disorder and PTSD (Table 1).

**Table 1.** Sample characteristics (n=15).

Characteristics	Participants
Sex, n (%)	
Male	11 (73)
Female	4 (46)
Race, n (%)	
Black	8 (53)
Hispanic or Latino	2 (13)
Native American	1 (6)
Non-Hispanic White	4 (26)
Age (years), n (%)	
30‐49	4 (26)
50‐64	8 (53)
65+	3 (20)
Diagnosis, n (%)	
Schizophrenia spectrum	5 (33)
Bipolar	6 (40)
PTSD^a^	4 (26)
2 Diagnoses	8 (53)
Education	
Less than high school graduate	1 (6)
High school graduate or general educational development equivalent	2 (13)
Some college	8 (53)
Bachelor’s degree	4 (26)
Marital status, n (%)	
Partnered	4 (26)
Not partnered	11 (73)

a PTSD: posttraumatic stress disorder.

### Emergent Themes and Subthemes of Attitudes Toward Wearable Devices From Qualitative Data

#### Attitudes Toward Wearable Devices

The interviews show that, out of the 15 participants, none had privacy concerns about using wearable mobile-monitoring devices that accessed their location or collected PGHD. Many of the participants expressed that their lack of concern over privacy issues was due to their belief that the personal health information being collected was common health information. One Veteran shared, “It was basic information, your health information… it’s just basic stuff.” Several participants also noted that many devices they already use, such as their cell phones, can track their location, so using a wearable device with that capability did not raise any concerns. In fact, many individuals expressed that they already expected the wearable devices to have location tracking capabilities when asked if they would like to participate in this portion of the project. In addition, some participants noted that they felt like they frequented the same locations and did not have reservations about that information being collected. “The data you would collect is my activities and that’s not a big secret,” stated one Veteran (Table 2).

Most of the participants interviewed stated that they liked the wearable devices, sharing that they were informative about their daily habits, relatively easy to use, and had a long battery life. For example, one Veteran said, “I think it was very nice, especially for a non-computer-literate person like myself. It was very easy to operate and stuff,” while another noted, “It gives you the important information.”

**Table 2.** Representative quotes. Attitudes toward mobile-sensing apps

Themes and subthemes	Quotes
Wearable monitoring benefits	
Sleep	“My favorite part is the sleep... you can compare it for the week or the month, and you can kind of track like where you’ve been… and what was helpful...”
Motivation	“It made me get out of my shell more and become more active. I would say it’s increased my quality of life.” “It kinda guides you. Kinda challenges you to do better the following week.” “... It showed me that my activity was increasing, and it motivated me more, to do more.”
Physical health	“My main thing right now is exercisin’ and that seems kind of simple but the steps that it counts that’s real important to me because I have a goal of how many steps to take.”
Ease of passive monitoring	“You can wear it and not even know that it’s there.” “The benefit is that it’s intuitive… I don’t have to input data. It does it automatically on its own, and that’s a convenience.”
Mental health tracking	“I can check in and let the Fitbit know my mood.” “It lets me know that I’m in good range, so that tells my mental health not to worry or have anxiety. So it keeps me in a good mood.”
Greater understanding of health	“If you feel differently that day, that can help you understand what may be causing you to have more mental difficulties that day and help you kinda narrow down what might be triggering you or causing additional difficulties.” “It lets me know how I’m doing in the moment... I can see how I’m doing health-wise.”
Device or app improvements	
Physical improvements	“I have a hard time seeing what’s on it because it doesn’t light up as well as it does at night..." “... the material of the watchband itself could cause irritation to some people..." “I would love a bigger face on it…”
Alerting help	“It can give a warning sign to whoever... and then they say, okay, dad is in a situation, or my loved one is in a situation. Let’s go talk to him and see if we need to talk him off a ledge.” “When you had some type of struggle in your mental health like maybe when you were feeling activated or when your heartrate was too high if it also vibrated then to let you know to kind of pay attention to the emotional side of things.”
Increased tracking capabilities	“It would be nice if I could put a reminder for me takin’ my medication.”
Greater app interaction	“Kinda like if it popped up... once an hour or however often instead of you having to remember, oh, I gotta go log this in. Especially if it detects something, if it would give you a notification. Hey, we noticed this.”
Wearable monitoring challenges	
Adjusting to wearing device	“It’s been really hard to remember to put it on..."
Lack of technical support	“I didn’t understand it… more technology I need to try to figure out.” “I really didn’t realize that the app, the phone app, had all these amenities to it.”
Difficulty understanding data	“And I don’t quite understand the sleeping portion of it because it’s just hard for me to understand it.”
Monitoring accuracy unclear	“… I don’t know whether I can depend on what that’s saying as far as my heart rate.”
Difficulty tracking emotions	“... I don’t think it has the capability to deal with... mental tracking as far as necessarily like mood and things like that.” “It will recognize stress level but not anxiety level.” “I guess the only thing it wouldn’t be able to address is the onset of the PTSD^a^.”
Attitudes toward wearable devices	
Like wearable device	“I think it was very nice, especially for a non-computer-literate person like myself. It was very easy to operate and stuff.” “It’s interactive, it’s very perceptive, and it gives me a ballpoint park of my health measures.” “It gives you the important information.” “It kinda shows you what’s going on with your health... it’s been a remarkable tool.”
Frequency of use - high	“... for the most part, it’s on all the time.”
Frequency of use - low	“I just don’t find it very user friendly.”
VA^b^ continued use	“I liked it. I recommend it to other guys.” "I’d continue to use it because it keeps me moving... I’m doing good with steps per day, and it shows significant improvement to my health.”
Privacy concerns - no	“... the data you would collect is my activities and that’s not a big secret.” “Because it was basic information, your health information… it’s just basic stuff.”
Attitudes toward mobile-sensing apps	
Like app	“... I like all the little categories and whatnot. You know? Helps me keep up with what the heck I’m doin’ and what’s goin on...” “I actually like it. It helps me keep track of my walking and just sitting down.” “... It’s got mindfulness where I can center myself, say, if I’m havin’ a bad day. I can go into this app. I can breathe, I can listen to somebody, positive thinking, and then I can come back.”
App usability - easy	“… I love how easy it is to use. The accessibility of it… it’s just really convenient.”
App usability - difficult	“It was kinda confusing. I’m still learning half the stuff that I’m reading.”
Clinician share	“I could bring this up at the physical and show it to the doctor, and that way give them some areas to look at... it would help with the physical and general well-being.” “It helps if that information is shared with our doctors or our psychiatrists. Kind of shows them a diagram of our mental health.”

a PTSD: posttraumatic stress disorder.

b VA: Department of Veterans Affairs.

Additionally, many participants shared that they believed the devices offered a comprehensive look at their health and overall well-being. “It’s interactive, it’s very perceptive, and it gives me a ballpoint park of my health measures,” stated one Veteran, with another adding, “It kinda shows you what’s going on with your health… it’s been a remarkable tool.” Some individuals shared that they were wearing and checking the data on their watches daily, “… for the most part, it’s on all the time,” said one Veteran. However, two participants noted that it was difficult to remember to put the device on in the mornings, while another shared, “I just don’t find it very user friendly,” both of which led to less frequent use.

The majority of participants agreed that they would continue to use the wearable devices if they were available through the VA. One Veteran shared, “I’d continue to use it because it keeps me moving… I’m doing good with steps per day, and it shows significant improvement to my health,” while another stated, “I liked it. I recommend it to other guys.”

#### Perceived Benefits of Wearable Devices

Many participants shared that they experienced a variety of benefits from using wearable mobile-sensing devices. All individuals interviewed stated that they were actively using the watches to track some aspect of their physical health, with their heart rate and steps taken per day ranking among the most popular categories. “My main thing right now is exercisin’ and that seems kind of simple but the steps that it counts that’s real important to me because I have a goal of how many steps to take,” expressed one Veteran. Findings also showed that nearly all participants were using the wearable devices to help monitor their sleep to gain information on the quality and duration of rest they were getting each night. One Veteran shared, “My favorite part is the sleep… you can compare it for the week or the month, and you can kind of track like where you’ve been… and what was helpful...”

Numerous individuals stated that using the wearable devices and seeing their PGHD, such as step count and overall activity, motivated them in a variety of ways. For example, several participants shared that after using the devices they felt motivated to do more physical activity (“…It showed me that my activity was increasing, and it motivated me more, to do more.”), leave their homes more often (“It made me get out of my shell more and become more active. I would say it’s increased my quality of life.”) and set healthier goals each week (“It kinda guides you. Kinda challenges you to do better the following week.”). In addition, nearly all Veterans shared that they felt that passive monitoring from wearable devices was a convenient and helpful way to gather their personal health information, sharing thoughts such as, “You can wear it and not even know that it’s there,” and “The benefit is that it’s intuitive… I don’t have to input data. It does it automatically on its own, and that’s a convenience.”

The majority of participants interviewed noted that they also used the watches to track aspects of their mental health, such as their stress and anxiety levels, which were reflected on the device’s accompanying cell phone app. For example, one Veteran expressed, “I can check in and let the Fitbit know my mood,” while another stated, “It lets me know that I’m in good range, so that tells my mental health not to worry or have anxiety. So, it keeps me in a good mood.” In addition, two individuals also shared that they were using the wearable devices to help manage their weight by monitoring the amount of calories burned per day, while 2 female Veterans interviewed stated that the watches allowed them to monitor their menstrual cycle effectively and conveniently.

Finally, most participants expressed that using the wearable devices helped give them a greater understanding of their overall health by allowing them to look over their sleep, stress, and daily activity data monitored by the devices. “If you feel differently that day, that can help you understand what may be causing you to have more mental difficulties that day and help you kinda narrow down what might be triggering you or causing additional difficulties,” stated one Veteran, while another noted, “It lets me know how I’m doing in the moment… I can see how I’m doing health-wise.”

#### Challenges Experienced With Wearable Devices

During the interviews, participants also mentioned some challenges to using the wearable devices. Nearly half of the individuals interviewed noted difficulties adjusting to wearing the watches due to either the wristband of the device being uncomfortable or difficulty remembering to wear the device. “It’s been really hard to remember to put it on…,” stated one Veteran. Participants shared that these challenges of remembering to wear the device typically occurred when they either took the watch off to charge or to sleep.

In addition, about half of the participants expressed that there were some technical difficulties in learning how to use the wearable device itself, as well as the accompanying phone app, which led to decreased use. “I didn’t understand it… more technology I need to try to figure out,” shared one Veteran. Individuals stated that this lack of comfort with and understanding of the technology particularly limited their use of the partner app, which contained elements such as sleep and stress information, charts of health data collected over time, as well as guided meditations and physical exercises. Many participants interviewed were unable to use these elements due to this barrier. “I really didn’t realize that the app, the phone app, had all these amenities to it,” noted one Veteran.

Some participants stated that they had some difficulty in understanding the significance of the PGHD collected from the wearable devices (“I don’t quite understand the sleeping portion of it because it’s just hard for me to understand it.”), and that further technical support or guidance on how to use the wearable device and the partner app would have been beneficial for their understanding and user experience. Also, many individuals expressed concern over the accuracy of the data collected. For example, some participants questioned the reliability of information (“… I don’t know whether I can depend on what that’s saying as far as my heart rate.”) and pointed out that some activity is unaccounted for when they are not wearing the devices, such as when they take them off to charge or sleep. Furthermore, some participants shared beliefs that wearable devices have limited capabilities when it comes to tracking certain aspects of their mental health, such as their emotions (“… I don’t think it has the capability to deal with… mental tracking as far as necessarily like mood and things like that.”), anxiety levels (“It will recognize stress level but not anxiety level.”), and other symptoms related to their SMI diagnosis, such as the onset of a manic or depressive episode (“I guess the only thing it wouldn’t be able to address is the onset of the PTSD.”).

#### Suggestions for Improvement of Wearable Devices

Participants also offered several suggestions on how to improve the wearable devices to increase their use and understanding of them, as well as improve their overall user experience. Individuals shared that some physical device improvements, such as adjustable screen brightness (“I have a hard time seeing what’s on it because it doesn’t light up as well as it does at night...”), a larger device screen (“I would love like a bigger face on it...”), and more comfortable wristbands (“… the material of the watchband itself could cause irritation to some people...”) would enhance their experience. About half of the participants expressed a desire for the devices to have increased tracking capabilities. Some suggestions for additional monitoring areas include pain levels, emotions, different forms of stress, and body temperature. In addition, individuals also expressed interest in the devices tracking medication adherence. “It would be nice if I could put a reminder for me takin’ my medication,” shared one Veteran, while others shared desires in wearables tracking their blood sugar and time spent on social media.

Many of the participants interviewed communicated a desire for increased interaction with the accompanying cell phone app. Some proposed that the app could be improved by providing additional exercise programs, sending medication reminders, and having an emotions log for individuals to check in on their mental health. “Kinda like if it popped up… once an hour or however often instead of you having to remember, oh, I gotta go log this in. Especially if it detects something, if it would give you a notification. Hey, we noticed this,” suggested one Veteran.

Additional suggestions included the watches vibrating or sending a phone notification to the individual if their heart rate or stress levels were too high, or even alerting help by sending a message to an individual’s clinician or family members under certain circumstances that may indicate a cause for concern. “When you had some type of struggle in your mental health like maybe when you were feeling activated or when your heart rate was too high if it also vibrated then to let you know to kind of pay attention to the emotional side of things,” said one Veteran, while another shared, "It can give a warning sign to whoever… and then they say, okay, dad is in a situation, or my loved one is in a situation. Let’s go talk to him and see if we need to talk him off a ledge.”

#### Attitudes Toward Mobile-Sensing Apps

Nearly all the participants stated that they liked the wearable’s partner cell phone app, many sharing that the app gives them a greater understanding of their overall health than just the wearable device alone, as the face of the watch itself only displays limited PGHD. “I actually like it. It helps me keep track of my walking and just sitting down,” stated one Veteran, while another said, “… I like all the little categories and whatnot. You know? Helps me keep up with what the heck I’m doin’ and what’s goin’ on...”

Most of the individuals who expressed liking the app noted that they were primarily using it to look at their step count, heart rate, and other PGHD from the wearable device in a longitudinal format displayed in graphs on the app dashboard. Participants stated that these graphs allowed them to see a visual representation of their health behaviors over the course of several weeks. In addition, two individuals expressed that they were using the mindfulness and physical exercise features included in the app and that they found these elements helpful for both their mental and physical health, with one saying, “… It’s got mindfulness where I can center myself, say, if I’m havin’ a bad day. I can go into this app. I can breathe, I can listen to somebody, positive thinking, and then I can come back.” Twelve participants stated that they had looked at the app at least once during the several weeks they were using the wearable devices, with many checking it daily or weekly. However, some of those individuals shared that they did not frequently open the app and relied more heavily on the watch itself to check their PGHD.

In addition, most participants noted that they felt the app was user-friendly due to the display buttons and categories of health information being simple and easy to understand. “… I love how easy it is to use. The accessibility of it… it’s just really convenient,” stated one Veteran. However, some participants expressed difficulty using the app. One Veteran noted, “It was kinda confusing. I’m still learning half the stuff that I’m reading,” with many others sharing that they felt the app was complicated to operate, that there was a lack of clarity about the meanings of the display categories, that they did not understand how their manually input data, such as daily food and water intake, could be used, and that checking the cell phone app was less convenient than looking at the watch itself.

Additionally, all but one individual interviewed agreed that they would feel comfortable sharing their PGHD with their clinicians to help inform and improve their health care treatment. Several shared thoughts such as, “It helps if that information is shared with our doctors or our psychiatrists. Kind of shows them a diagram of our mental health,” and “I could bring this up at the physical and show it to the doctor, and that way give them some areas to look at… it would help with the physical and general well-being,” of patient health and treatment.

## Discussion

### Principal Results

Participants with SMI in this sample generally held positive perceptions toward wearable mobile-monitoring devices and were able to incorporate wearing the health and fitness trackers into their daily lives, many noting that the passive data collection was convenient. Qualitative data showed that participants used the devices to track various aspects of their health, such as sleep, heart rate, stress, and physical activity, and found that devices gave them a greater understanding of their overall health. Some participants experienced technical difficulties when using the wearables and their partner phone app, which may have limited their use of the devices. Additionally, participants suggested that some physical changes to the wearable devices, such as screen brightness and screen size, would increase usability and accessibility of these devices.

### Comparison With Prior Work

The majority of participants enjoyed their experience using the mobile-sensing watches and accompanying app, which is consistent with previous research [33,34]. Although some studies have found privacy concerns about personal health data collection and location tracking to be potential barriers to using mobile-monitoring devices among the SMI population [35], qualitative data revealed that these concerns were not present for Veterans in the study.

The convenience and capability of wearable devices to monitor PGHD may be particularly helpful for individuals with SMI, as members of this population often experience psychological barriers to physical activity and other healthy lifestyle choices, which can have negative impacts on their overall well-being [36]. Interviews revealed that mobile-sensing devices can help address this challenge by increasing participants’ levels of motivation to engage in physical activity from week to week upon seeing their PGHD, such as their daily step count, collected by the wearable devices. Individuals also reported that they appreciated the ease and convenience of passive monitoring and that the devices helped increase their understanding of their overall health, suggesting that incorporating these devices into participants’ daily lives is both feasible and beneficial.

The qualitative reports also brought to light some challenges of using mobile-monitoring devices. Some physical aspects of the design of the watches, such as the buttons on the device being difficult to operate, became barriers to use for several of the participants. Individuals also shared that wristbands being uncomfortable led to some challenges keeping the devices on for extended periods of time, especially during sleep, and that the lack of brightness of the watch face screen made it difficult for them to see their data when outside during the day. Additionally, differing comfort with using technology made it particularly difficult for several participants to use the device’s companion cell phone app, which has previously presented challenges of wearable device use for this population [37]. In future research, more detailed training with participants upon initially receiving the wearable device, as well as ongoing technical support during device use, may be beneficial to ensure individuals are able to access all PGHD and amenities provided by these devices. In addition, using peer support coaches to help with mobile health interventions has been shown to be helpful for Veterans with SMI [38], and these active supports may also be beneficial to increasing the accessibility and understanding of mobile-sensing devices for this population.

### Limitations

This study has some potential limitations. In this sample, we interviewed Veterans with SMI receiving treatment at the GLA VA, an urban area. These individuals volunteered to participate in a study involving the use of wearable devices that track their physical activity and location, and therefore, the thoughts and experiences of these participants may differ from those of other individuals who did not consent to participate and use these devices. Secondly, the interview guide was informed by the Technology Acceptance Model, which did not explore differences in participants’ thoughts or experiences based on sex, race, or age, and potential differences in these areas did not come up organically during interviews, which limits our understanding of how these characteristics may influence perceptions of wearable devices for individuals with SMI. Further study of wearable devices that explore the influences of sex, race, and age is needed to expand our understanding of the perceptions and uses of wearable devices in this population. In addition, although access to a smartphone was not a barrier for any participants in our sample, and research has shown smartphone use in individuals with SMI to be as high as about 60% [18], this obstacle may be present for some individuals. Fitbits can be activated through a smartphone, tablet, or computer, and are able to collect PGHD, such as steps, heart rate, and sleep information, without needing further access to additional technology. However, to use their full amenities, such as seeing long-term data trends and using GPS, the device needs to be synced to the Fitbit app via smartphone or tablet. Health care facilities, such as the VA, may be able to reduce potential technological accessibility barriers through programs aimed at reducing this digital divide, such as via the Office of Connected Care. Finally, participants’ duration using the devices varied from periods of two weeks to about a month. Individuals who had been using the watches for the shorter period had less time to engage with the device and accompanying app, which may have led to decreased PGHD collection and allowed less time for participants to become acquainted with using the device or to experience potential challenges.

### Conclusions

Research has increasingly turned to the use of technology to help address barriers of PGHD and communication between patients and providers. Using mobile-monitoring devices and PGHD to improve treatment outcomes for individuals with SMI has been gaining traction, especially as more research uncovers its potential to benefit patients in this population. These devices conveniently collect passive sensor data that can be shared with health care organizations to help fill in gaps on important health trends that patients may experience between medical visits, such as stress levels, physical activity, and sleep quality and duration. Through using these devices, patients could potentially self-monitor their activities and identify changes in their behavior that may be associated with a lapse in mental health. In addition, these devices can provide ample information about various health behaviors that could be used to prevent relapse and modify approaches to patient care. This study indicates that wearable devices should be feasible overall for participants with SMI and that individuals within the population are accepting of these devices. While there may be some unique challenges in this population, incorporating wearable devices into patient care could provide an opportunity to enhance treatment outcomes and prevent relapse. With greater emerging technologies that monitor mental and physical health needs, there is a promising future for the use of wearable devices to support this population.

## Abbreviations

GLA VA: Greater Los Angeles Department of Veterans Affairs
PGHD: Patient-generated health data
PTSD: posttraumatic stress disorder
SMI: serious mental illness
VA: Department of Veterans Affairs
